# Proceedings of the Patient Reported Outcome Measures (PROMs) Research Conference, Sheffield 2021: 16–17 June 2021, Virtual, Sheffield, UK

**DOI:** 10.1007/s11136-022-03096-0

**Published:** 2022-03-01

## Introduction

### Proceedings: PROMs research (virtual) Conference, University of Sheffield, 16th & 17th June 2021

Tracey Young^1^, Jill Carlton^1^, Phil Powell^1^, Laura Flight^1^, Alexis Foster^1^, Lucy Musson^1^, Amanda Lane^1^, Kathryn Mackellar^1^, Patricia Holch^2^, Grace Turner^3^.

^1^University of Sheffield, Sheffield, United Kingdom. ^2^Leeds Beckett University, Leeds, United Kingdom. ^3^University of Birmingham, Birmingham, United Kingdom.

The 5th UK Patient Reported Outcome Measures (PROMs) Research Conference (#UKPROMS) held on the 16^th^ and 17^th^ June 2021 was attended by 154 academics, clinicians, PhD students and patients, representing 105 organisations. As the conference was held online, due to the Covid-19 pandemic, it attracted an international audience, including delegates from the United States, Canada, Australia, Europe, Hong Kong, Jordan and Africa. The aim of the conference was to bring together leading experts and early career researchers to engage in the latest advances in the field of PROMs research and implementation. The conference focused on researcher-led activities on methodologies around the development, testing and use of PROMs in different contexts and settings. The conference made a return to Sheffield having previously been hosted by the Universities of Oxford, Birmingham and Leeds Beckett.

## Conference summary

Due to the Covid-19 pandemic and UK Government restrictions the event was held online and was structured around a mixture of facilitated plenary sessions, workshops, parallel sessions and poster displays by PROMs researchers from across the UK and internationally. There were 27 oral presentations and 36 posters displayed, with two plenaries and two workshops with invited speakers.

The oral presentations included *Young people and rare conditions*; *Methods—Developing or refining PROMs*; *Implementing PROMs in health care*; *Methods – Measuring what matters*; *Measuring outcomes in different groups of settings*; *Methodological issues in instrument development*; *Social care*; *Patient, public involvement and engagement*; *Methods-Psychometrics/item response theory*.

**Dr Donna Rowan**, from ScHARR, gave a presentation on the challenges and emerging evidence on measuring and valuing health in children for economic evaluation. This was followed by a panel of experts, **Professor Nick Bansback**, **Dr Clara Mukuria**, **Dr David Wellsted** and **Dr Alexis Foster**, chaired by **Professor John Brazier**, answering PROMs ‘Hot Topic’ questions from delegates.

Two parallel workshops were held.

**Lucy Musson**, University of Sheffield, **Sharon Caunt**, Sheffield Teaching Hospitals NHS Foundation Trust, **Philly Hare**, Innovations in Dementia and **Angus Houston**, Public/Patient Participant, Dementia Enquiries project, shared learning from people with dementia in the driving seat of research.

**Professor Nick Bansback**, School of Population and Public Health, University of British Columbia, Canada, examined how routine PROMs can be used in clinical practise to help patients and their clinicians make informed, shared decisions.

The conference offered three prizes which were judged and awarded as follow:

- Best PhD poster: **Elke Rammant**, Ghent University, Ghent, Belgium and King’s College London, United Kingdom
- Patient and Public Involvement Team Award, ‘Best PPIE team who have demonstrated—excellence/and/or innovation in involving the public – oral or poster’: **Laura Gillis**, Sheffield Teaching Hospital, Sheffield, United Kingdom
- Early Career Researcher Award—‘Best Oral Paper’. Sponsored by Oxford University Innovation: **James Glasbey**, NIHR Global Health Research Unit on Global Surgery, University of Birmingham, United Kingdom

## Ethics declarations

Conflict of interest: There is no conflict of interest regarding the research, authorship, or publication of this article.

Consent for publication: Informed consent was obtained.

## Abstracts for oral presentations

### Session 1a: Young People and Rare Conditions

#### A1 Psychometric and measurement invariance properties of a novel outcome measure for children with life-limiting and life-threatening illnesses

Eve Namisango ^1,2^, Fliss EM Murtagh ^2,3,4^, Katherine Bristowe ^2,4^, Julia Downing ^5^, Richard A Powell ^6^, Mackuline Atieno ^1^, Zipporah Ali ^7^, Faith Mwangi Powell ^8^, Irene J Higginson ^2,4^, Richard Harding ^2,4^. ^1^African Palliative Care Association, Kampala, Uganda. ^2^Cicely Saunders Institute, London, United Kingdom. ^3^Wolfson Palliative Care Research Centre, Hull York Medical School, University of Hull, Hull, United Kingdom. ^4^Florence Nightingale Faculty of Nursing, Midwifery and Palliative Care, King’s College London, London, United Kingdom. ^5^International Children’s Palliative Care Network, Pretoria, South Africa. ^6^Imperial College London, London, United Kingdom. ^7^Kenya Hospices and Palliative Care Associations, Nairobi, Kenya. ^8^Girls Not Brides, London, United Kingdom.

Aims: To determine the validity, reliability, measurement invariance, acceptability, and interpretability of the novel Children’s Palliative Care Outcome Scale (C-POS). Methods: Multi-level, mixed-methods, study conducted at tertiary care centres in four African countries. 1) Qualitative: interviews exploring symptoms, concerns and outcomes informed content validity, through item-theme mapping and panel of experts review for face validity assessment. 2) Quantitative: Children (0-17 years) receiving palliative care and their carers were recruited for discriminant validity, reliability, measurement invariance, acceptability, and interpretability. Children/carers completed the C-POS at four time points. Classical test and item response theory methods were utilised to establish construct validity, internal consistency, measurement invariance, test–retest reliability, responsiveness, acceptability, and minimum important difference. Results: 1) 120 interviews were conducted (children n = 59, carers n = 61). Face and content validity was demonstrated as the C-POS items mapped to qualitative interview themes 2) 434 children were recruited for quantitative data collection and of these 302 participated in the repeated measures component (response rate 94%), and 92% completed the four C-POS measurements. The two-factor structure (child and family) was confirmed along with discriminant and known groups validity and construct equivalence for both versions. Controlling for age there was no differential-item functioning by country. The test characteristic curve information confirmed the moderate internal subscale internal consistency scores between 0.3 to 0.9 for the proxy version and 0.3–0.5 for the self-report version. Test–retest reliability was hypothesised for all items with weighted kappa range for scores of self-reports (0.43–0.57), proxy versions (0.35–0.64), and family items (0.51–0.71). Responsiveness was demonstrated as hypothesised. Median completion time at the last visit was 10 min for both versions. The minimum important difference was 3 for the self and proxy report versions on a scale of 0–30 and 4 for the child and family scale on a scale of 0–55. Conclusions: With minor adaptation, the C-POS can measure person-centred outcomes in paediatric palliative care, with robust psychometric properties. The study provides a breakthrough on outcome measurement in paediatric palliative care. Disclosures: The authors declared no competing interests.

#### A2 “if they knew what I thought was important …that would help a lot more”: Methods for involving children and young people with life-limiting and life-threatening conditions in development of a paediatric palliative care outcome measure.

Debbie Braybrook ^1^, Lucy Coombes ^1^, Anna Roach ^1^, Hannah Scott ^1^, Daney Harðardóttir ^1^, Katherine Bristowe ^1^, Clare Ellis-Smith ^1^, Michelle Hills ^2^, Linda Maynard ^3^, Anna-Karenia Anderson ^4^, Eve Namisango ^1^,^5^, Irene Higginson ^1^, Julia Downing ^6^, Myra Bluebond-Langner ^7^, Lorna Fraser ^8^, Fliss E M Murtagh ^9^, Richard Harding ^1^.

^1^Cicely Saunders Institute of Palliative Care, King’s College London, London, United Kingdom. ^2^Martin House Hospice and Leeds Teaching Hospitals NHS Trust, Leeds, United Kingdom. ^3^East Anglia’s Children’s Hospices, Cambridge, United Kingdom. ^4^Royal Marsden Hospital, London, United Kingdom. ^5^African Palliative Care Association, Kampala, Uganda. ^6^International Children's Palliative Care Network, Cape Town, South Africa. ^7^Louis Dundas Centre for Children's Palliative Care, University College London, Great Ormond Street Institute of Child Health, London, United Kingdom. ^8^Martin House Research Centre, University of York, York, United Kingdom. ^9^Wolfson Palliative Care Research Centre, Hull York Medical School, University of Hull, Hull, United Kingdom.

Background: Meaningful involvement of children and young people (CYP) in health research aligns with the UN Convention on the Rights of the Child. Studies collecting primary data from CYP with life-limiting and life-threatening conditions, that could inform content validity of outcome measures, are challenging and rare. Aims: To advance methods for meaningful involvement of CYP in the development of paediatric palliative care outcome measures. Methods: Solutions to challenges in data collection were iteratively tested and refined. CYP involvement was achieved through: 1) Semi-structured qualitative interviews. 26 CYP aged 5–17 with life-limiting and life-threatening conditions across 9 clinical sites, 2) Repeated engagement with Young Person’s Advisory Group. Results: CLINICIANS: To achieve CYP recruitment, strong links developed through initial site visits for partnership building, one team member allocated clinical site contact, offered conference scholarships, summary sheet addressed concerns about introducing study to families, and quarterly newsletter. FAMILIES: To encourage consent of parent/caregivers to CYP participation, described interview topics and wording during pre-interview discussions, meeting families pre-interview where possible. CYP: Developmentally appropriate play/chat preceded CYP assent and utilised to re-engage and alleviate tension; interviews began with talk about likes and dislikes; examples used to facilitate discussion; interviews paused where required to account for medical needs; caregiver input for support and interpretation where necessary. YOUNG PERSON’S ADVISORY GROUP: Partnership building with leader of a young person’s advisory group, remote meetings to sustain engagement during the COVID-19 pandemic, developmentally appropriate content, feedback to CYP. Conclusions: CYP are rarely engaged to inform content validity in outcome measure development. By responding to the needs of each stakeholder group involved in enabling CYP participation, we meaningfully engaged a traditionally ignored population. The methods developed here offer lessons for future research for CYP with complex health needs. Acknowledgement: Submitted on behalf of C-POS consortium. Disclosures: The authors declared no competing interests.

#### A3 Indictors of Quality of Life (QoL) for children with Goldenhar Syndrome: Exploring parental views.

Patricia Holch ^1^, Rebecca Hitchen ^2^, Georgina Jones ^1^.

^1^Leeds Beckett University, Leeds, West Yorkshire, United Kingdom. ^2^Goldenhar UK, Charity, Stoke on Trent, Staffordshire, United Kingdom.

Background: Goldenhar is a rare congenital syndrome (1: 5600 births) with no known cause, resulting in a wide range of soft tissue and bone impairments including vertebrae displacement mainly affecting one side of the body. Children with Goldenhar Syndrome often display the underdevelopment of an ear, jaw and cheek, malformations of the middle-ear canals, hearing loss, eye irregularities, large skin tags, cleft lip or wide mouth, club foot and congenital hip dislocation. Although the clinical manifestations have been researched, few studies have explored QoL and psychosocial wellbeing. Aim: The aim was to gather initial QoL indicators from parents, with the ultimate aim of developing a future Patient-Reported-Outcome-Measure (PROM). Methods: A volunteer sample of five male and female (n = 10) parents who have a child with Goldenhar Syndrome took part in semi-structured audio recorded interviews at the Goldenhar UK Charity family conference in 2018. Interviews were transcribed verbatim and subject to thematic analysis (Braun & Clarke 2006). Results: Findings exposed the challenges children face and indicated that impaired QoL was an issue. Parents revealed a need for ongoing emotional support for them and their children. Depression and anxiety were prevalent as were parental guilt and profound uncertainty. They described that their children often felt stigmatised and isolated due to a lack of representation and acceptance within society. More positive findings were the peer support and coping strategies for both parents and children enabled by the Goldenhar UK charity. Conclusions: We have started to take a family systems approach to understand the challenges of Goldenhar Syndrome by initially interviewing parents. Clear barriers to QoL include: depression, anxiety, parental guilt, isolation, and impact on relationships. The next stage includes gathering further QoL information from children and young adults with Goldenhar Syndrome to ensure face and content validity in the development of a forthcoming PROM. References: Virginia Braun & Victoria Clarke (2006) Using thematic analysis in psychology, Qualitative Research in Psychology, 3:2, 77–101, https://doi.org/10.1191/1478088706qp063oa. Disclosures: The authors declared no competing interests.

### Session 1b: Methods – Developing or Refining PROMs.

#### A4 Development of a new generic, modular patient-reported resource-use measure (ModRUM).

Kirsty Garfield, Joanna Thorn, Sian Noble, Samantha Husbands, Will Hollingworth.

University of Bristol, Bristol, United Kingdom.

Background: Patient-reported resource-use measures (RUMs) are designed for capturing resource-use data in economic evaluations of healthcare. RUMs are commonly developed on a trial-by-trial basis, with validation rarely performed. We are developing a standardised modular RUM (ModRUM) from items identified in a Delphi survey. ModRUM includes a core healthcare module and optional depth-adding questions if more details are required. Aims: To develop and test the validity (face, content, construct and criterion), acceptability and feasibility of ModRUM. Methods: With reference to existing RUMs, questions were formulated from Delphi items. Four evaluation studies were conducted, with modifications made between and within studies: (1) experienced health economists participated in qualitative interviews; questions focused on item relevance, clarity, conciseness and omission; (2) ‘think-aloud’ interviews with retrospective probing were conducted to assess patient comprehension and acceptability; (3) health economists piloted the adaptation process for recently funded trials and completed a feedback survey; (4) ModRUM is being piloted with primary care patients; responses will be compared with medical records. Results: (1) 10 health economists were interviewed. Most core questions were judged adequate. Feedback varied on the granularity of depth questions. (2) 20 patients were interviewed. 80% of questions were completed without issue and responses were consistent with what questions intended to measure. (3) 11 health economists participated; most reported that adaption was feasible and that they would use ModRUM, over half made adaptations that were not specified as permitted. (4) To date, 80 patients have participated. Recruitment will conclude in March 2021. Conclusions: ModRUM is a new standardised generic RUM. The validity (face and content) and acceptability have been demonstrated. ModRUM includes adaptable elements to encourage uptake and ensure relevance in all trials. Health economists have reported that ModRUM is feasible to adapt and suitable for trials. Other measurement properties will be assessed in the patient pilot. Disclosures: The authors declared no competing interests.

#### A5 Identifying Key Components of a Healthy and Positive Pregnancy and Birth

Ekaterina Bordea, Rachael Hunter.

University College London, London, United Kingdom.

Background: Patient Reported Outcome Measures are rarely used in evaluating maternal health services. The most common questionnaire used in economic evaluations, EQ-5D, does not reflect what is important to women during pregnancy, such as quality of care or the 2018 WHO recommendation on the importance of a positive childbirth experience. Aims: Feasibility of identifying what women consider are the important attributes of a positive pregnancy and birth and asking them to rank them. Methods: Women in the REACH Pregnancy Circles feasibility trial and who had recently given birth were asked to be involved in a patient and public involvement (PPI) session to identify what outcomes were important to them as part of pregnancy, birth and postnatal care. Based on the attributes a purposely designed questionnaire was built using a web-based survey tool Opinio. The online questionnaire was distributed to mailing groups and advertised on social media platforms. Best–worst scaling and a Balanced Incomplete Block Design was used for ranking the attributes and to provide estimates of their weights. A sample size of 400 participants was required for comparison between groups (women who recently gave birth (< 12 months), medical professionals and general population). Results: Women in the PPI group identified the attributes identified are mother’s health, baby’s health, choice, feeling in control, provision of information, feeling supported, continuity of care and feeding support. It was feasible to ask women who recently gave birth and the general population to rank these attributes, but we struggled to recruit medical professionals. We report provisional estimates for weights. Conclusions: Women could identify the attributes most important to them as part of a positive pregnancy and were able to rank these attributes. Provisional estimates will be used in the REACH Pregnancy Circles trial, but further work is required to validate the attributes and weights. Trial Registration: ISRCTN ISRCTN66925258. Registered 03 April 2017. Disclosures: The authors declared no competing interests.

#### A6 Which quality of life domains are most sensitive to the recall period in individuals who experience fluctuations in their health?

Lidia Engel^1^, Natasha Hall^1^, Nikki McCaffrey^1^, Brendan Mulhern^2^, Long Le^1^, Cathy Mihalopoulos^1^, Kate Anderson^1^.

^1^Deakin University, Burwood, Australia. ^2^University of Technology Sydney, Sydney, Australia.

Background: Obtaining reliable quality of life (QoL) estimates in individuals with fluctuating health conditions is challenging, as symptoms often vary by time of the day, day of the week, or during the month. Besides the timing of the assessment, the recall period poses another important methodological consideration. Aims: The aim of this study was to explore which QoL domains are most sensitive to the recall period in individuals with fluctuating health conditions. Methods: Interviews were undertaken with individuals with multiple sclerosis (n = 6), epilepsy (n = 4), bipolar disorder (n = 5) and migraine (n = 11). At the start of each interview (via Zoom), individuals completed the AQoL-8D, the most comprehensive generic QoL instrument, which measures eight domains (independent living, senses, pain, mental health, happiness, self-worth, coping, and relationships) ‘over the past week’. Participants were instructed to think-aloud their thoughts while completing the questionnaire. Semi-structured interviews were then conducted to explore the appropriateness of the 7-day recall period in relation to fluctuating symptoms. Transcribed interviews were analysed thematically alongside a data extraction form that documented which domains were most sensitive to fluctuations. Results: The sample’s mean AQoL-8D score was 0.55 (bipolar disorder: 0.40; multiple sclerosis: 0.40; epilepsy: 0.66; migraine: 0.67). Independent living was the only domain that was sensitive to fluctuations and the chosen recall period across all four conditions. Pain was the second most sensitive domain, although less impacted by fluctuations in individuals with bipolar disorder, who on the other hand, reported that their responses would differ to happiness, mental health, and coping, depending on whether they are in a manic or depressive state. There were inconclusive findings for senses, relationships and self-worth. Conclusions: In conclusion, while not all QoL domains are equally impacted by fluctuations and hence the chosen recall period, there were variations across conditions, posing challenges to QoL assessment using generic QoL instruments. Disclosures: The authors declared no competing interests.

### Session 1c: Implementing PROMs in Health Care

#### A7 Embedding Patient-Report Outcome Measures in the Curriculum: Lessons Learned from Student Physiotherapy Education based on a Mixed Methods Study.

Lisa Edwards^1^, Angela Wolff^2^, Andrea Dresselhuis^2^, Jamie Moseley^1^.

^1^University of Bradford, Bradford, United Kingdom. ^2^Trinity Western University, Langley, British Columbia, Canada.

Aims: Substantial literature has highlighted the importance of patient-reported outcome and experience measures (PROMs / PREMs, respectively) to collect clinically relevant information from patients to better understand and address what matters to them. Data from PROMs / PREMs is critical to support clinical reasoning, judgement, and action in a person-centred approach. Implementation of patient-reported measures by clinicians in practice can be a struggle. We posit that embedding them into pre-registration education can facilitate their inclusion in the practice of post-registration, novice clinicians. This project builds on existing evidence about how clinicians implement patient-reported measures into routine practice by incorporating the perspective of physiotherapy students. Methods: Guided by a mixed methods approach, this study involved a systematic review to synthesize the peer-reviewed research evidence (qualitative and quantitative) from eight databases (2010–2020) (n = 170). An implementation science framework was used to identify the needs and individual factors influencing clinicians at the point of care. These findings were triangulated by qualitative data obtained from physiotherapy students at a UK higher education institute. Results: This presentation will highlight key findings that are similar to, and different from, both the published literature and students’ perspectives regarding the implementation of patient-reported measures. Opportunities for embedding patient-reported measures in curriculum and developing teaching strategies to support an emphasis on the patients’ needs will be discussed. Both students’ and providers’ experiences in applying patient-reported measures in practice will be shared with an emphasis on how they inform clinical reasoning, judgement, and action. Conclusions: The implementation of PROM / PREMs into practice requires careful consideration of integrating the education of their use into curriculum. Learning at a grass-roots level and being exposed pre-registration, provides a foundation to foster their use in daily practice. Furthermore, incorporating implementation science theory is crucial to address both the needs of and individual factors influencing students and clinicians. Disclosures: The authors acknowledge financial support for this project from the BC SUPPORT Unit, Patient-Centred Measurement Methods Cluster Award Number: PCM-004. The BC SUPPORT Unit receives funding from the Canadian Institutes of Health Research and the Michael Smith Foundation for Health Research. The authors declared no competing interests.

#### A8 Personalised PROM development in a rare cancer: a cancer nurses’ perspective

Jane Ireson^1^, ^2^, Patricia Holch^2^, Stephen Radley^1^, Diana Greenfield^1^, Matt Winter^1^, John Tidy^1^, Georgina Jones^2^.

^1^Sheffield Teaching Hospital, Sheffield, United Kingdom. ^2^Leeds Beckett University, Leeds, United Kingdom.

Background: Gestational Trophoblastic Disease (GTD) is a rare complication of pregnancy that can develop into cancer, where a traditional biomedical focus has meant little is known about the impact of GTD on women’s lives. The GTD nursing team in Sheffield have co-designed with patients an electronic PROM (ePAQ-GTD) that enables women to voluntarily self-report on the symptoms and impact of GTD in routine care. ePAQ-GTD replaces and extends the use of a long-standing nursing tool, the Holistic Needs Assessment (HNA) within GTD care. Aims: To summarise preliminary ePAQ-GTD data through engagement and feedback. Discuss the relationship between the HNA and PROM tools within cancer clinical practice. Methods: Mixed methods initial validation of ePAQ-GTD through the collection of engagement and Likert scale-based patient survey data, including free text comments. Results: EPAQ-GTD has replaced the HNA and has a high value and low burden to patients. During the first year, 402 (51%) ePAQ-GTD questionnaires have been completed by patients at different stages of GTD (72% screening, 28% chemotherapy). 85% (n = 53) of chemotherapy patients have engaged with ePAQ-GTD. In the postal survey, (N = 47) 87% agreed ePAQ-GTD is valuable in their care, 82% felt it had a low burden. Conclusion: PROMs are increasingly integrated into routine cancer care to collect aggregate QoL data to ensure a patient focus in research and clinical practice. National guidelines recommend HNA for all cancer patients to routinely identify a patient’s holistic needs (NHS England 2017). PROMs overlap with the remit of HNA and specialist cancer nurses have historically routinely collected PROM data through the HNA process, but this has not been captured as aggregate QoL data, nor are PROMS integrated routinely in clinical care. The role of the HNA and PROMs have evolved independently within cancer clinical practice. However their scope and utility are merging, as exemplified by the personalised PROM tool ePAQ-GTD. References: Achieving World Class Cancer Outcomes: A Strategy for England 2015–20. [Progress Report 2017] available from: https://www.england.nhs.uk/wp-content/uploads/2017/10/national-cancer-transformation-programme-2016-17-progress.pdf. Disclosures: The authors declared no competing interests.

A9 Published https://doi.org/10.48037/mbmj.v8i9.1285

### Session 2a: Measuring What Matters

#### A10 Published https://doi.org/10.1136/ANNRHEUMDIS-2021-EULAR.164

#### A11 Published https://doi.org/10.1007/s11136-021-02976-1

#### A12 Concept elicitation for setting-sensitive patient-reported outcome measurement in telemedicine: A context-related approach to an extended working model for quality of life assessment

Klara Greffin^1^, Holger Muehlan^1^, Neeltje van den Berg^2^, Wolfgang Hoffmann^2^, Oliver Ritter^3^, Michael Oeff^3^, Georg Schomerus^4^, Silke Schmidt^1^.

^1^University of Greifswald, Greifswald, Germany. ^2^University Medicine Greifswald, Greifswald, Germany. ^3^Brandenburg City Hospital, Brandenburg an der Havel, Germany. ^4^University Medicine Leipzig, Leipzig, Germany.

Background: Telemedicine (TM) can be an improvement in managing a disease and could have a positive impact on QoL. Yet, reviews show inconsistent results with regard to the effectiveness of TM on patient-reported outcomes (PRO) like quality of life (QoL). We assume that existing PRO measures may lack sensitivity to assess the intended outcomes of TM. Aims: Our study aimed to inform concept elicitation by exploring the experiences of TM and its impact on QoL from complimentary perspectives of patients with different health conditions (mental disorder vs. chronic disease) and different TM approaches (active vs. passive) as well as from TM professionals from diverse settings. Methods: Overall, 63 semi-structured single interviews and 15 focus groups with 68 participants have been conducted. Participants were patients with heart failure or major depression with or without TM as well as TM professionals. Content analysis was used to encode the qualitative data material. Results: The majority of aspects that influence the QoL of patients could be assigned to an already established working model of QoL However, some aspects that were deemed relevant were not covered by the pre-existing domains yet. Our category system comprised one additional domain with 4 facets and 35 attributes related to QoL issues in TM in particular. Therefore, we added this domain to the model, referred to as healthcare-related QoL. Conclusions: This qualitative study brought forth specific aspects of QoL evolving in TM. The results reinforce the assumption that existing QoL measures lack sensitivity to assess the intended outcomes of TM. We will address this deficiency by a TM-related extension of QoL assessment and the development of a suitable add-on PRO measure based on the resulting category system. This contributes to increase the inclusion of the patient perspective in health services research of TM. Disclosures: The authors declared no competing interests.

### Session 2b: Measuring Outcomes in Different Groups or Settings.

#### A13 Published https://doi.org/10.1007/s11136-020-02626-y

#### A14 Carer quality of life in cystic fibrosis: a comparison of preference-based measures

Sulayman Chowdhury^1^, David Mott^2^, Patricia Cubi-Molla^2^.

^1^City, University of London, London, United Kingdom. ^2^Office of Health Economics, London, United Kingdom.

Background: Many diseases involve a large burden of care on informal carers. One example is cystic fibrosis (CF), which is a rare severe condition in which the patients require considerable care, which is often provided by parents. Carer quality of life (QOL) impacts can be included in economic evaluations of new treatments. However, it is unclear whether commonly used preference-based measures are suitable in this context. Aims: This study aimed to assess the QOL of informal carers of patients with CF, and to compare the psychometric performance of two generic preference-based measures (EQ-5D-5L and ReQoL) and two carer-specific preference-based measures (CarerQol and ASCOT-Carer). Methods: An online questionnaire containing each measure along with additional questions was designed. Carers of patients with CF from the United Kingdom were invited by a carer organisation, CF Voices, to complete the questionnaire. The performance of the measures was primarily assessed using known group validity (based on CF severity) using analysis of variance and regression analysis. Results: A total of 258 carers completed the questionnaire. The majority were female (n = 219; 85%), highly educated (n = 213; 83%), and caring for patients with moderate CF (n = 147; 57%). Irrespective of the measure, mean utilities were lower when the severity of CF in the patient was higher. However, the mean differences were only statistically significant with ReQoL (10% level) and CarerQol (5% level). Regression analyses indicated that the carer-specific measures appeared to be sensitive in relation to caring variables (e.g., daily care hours) but were unable to distinguish between mild and moderate CF. The opposite was the case for the generic measures. Conclusions: Overall, each of the included measures performed relatively well. Whilst EQ-5D-5L is more commonly used in economic evaluations, the results suggest that ReQoL may perform better in this context. Disclosures: The authors declared no competing interests.

#### A15 Gender perspective in the use of patient-reported outcome measures (PROMs) in rare diseases.

Sara Pérez, Silvina Funes, Begoña Leyra.

Complutense, University of Madrid, Madrid, Spain.

Background: When referring to health conditions, we can find a wide range of diseases that can be associated with various impairments, limitations or restrictions. Knowing patients' perceptions is nowadays a gold standard to be able to plan therapeutic processes, health or social policies. Rare diseases are a heterogeneous group of diseases that show a low prevalence, high mortality and morbidity rates, and difficulties in access to diagnosis, treatment and research that represent a socio-health challenge. In turn, the pairing of women and disability is widely known in the scientific literature as a risk factor for potential health, educational and social disadvantages. This is why we consider that access to information on the perception of patients with rare diseases, and the development of the concept and utility for the clinician of the PROs (Patient Reported Outcomes) from a gender perspective, must be a priority in the improvement and advancement of an inclusive society. Aims: The aim of our work is to update the current knowledge on the use of the gender perspective in the field of PROs through a systematic literature review. Methods: We have carried out a systematic literature review taking into account parameters such as patient age, gender, pathology profile and its characteristics, as well as the professional profiles involved with these patients. Results: After analysing the data, the results we present show notable shortcomings with respect to the use of the gender perspective in PROs in a generalised manner. We found areas with greater development such as the medical specialties of gynaecology and oncology. Conclusions: We conclude that it is necessary to know and develop the PROS from a gender perspective. At the same time, we highlight the need for a more holistic vision, not only in terms of the clinical or rare disease diagnosed. For our conclusions we provide concrete work proposals. Disclosures: The authors declared no competing interests.

### Session 2c: Methodological Issues in Instrument Development.

#### A16 Combining expert judgment and psychometric information to create the Global Scale for Early Development (GSED).

Gareth McCray^1^, Melissa Gladstone^2^, Vanessa Cavallera^3^, Dana McCoy^4^, Marcus Waldman^5^, Iris Eekhout^6^, Stef van Buuren^7^, Magdalena Janus^8^, Patricia Kariger^9^, Maureen Black^10^, Ann Weber^11^. ^1^Keele University, Stoke on Trent, United Kingdom. ^2^University of Liverpool, Liverpool, United Kingdom. ^3^World Health Organisation, Geneva, Switzerland. ^4^Harvard University, Cambridge MA, USA. ^5^University of Nebraska, Lincoln, USA. ^6^University Medical Center Utrecht, Utrecht, Netherlands. ^7^Netherlands Organisation for Applied Scientific Research, Leiden, Netherlands. ^8^McMaster University, Hamilton, Canada. ^9^University of California, Berkeley, USA. ^10^University of Maryland, College Park, USA. ^11^University of Nevada, Reno, USA.

Background: The Global Scale for Early Development (GSED) a global and culturally neutral measure of child development under 3 years of age is designed to allow comparisons within and across countries. The project has been undertaken by an experienced team of international researchers led by the World Health Organisation. Ultimately, the GSED will comprise two tools anchored on the same scale: firstly, a caregiver reported measure aimed at population level monitoring, and secondly, a direct assessment tool to be used for programmatic evaluation. Aims: The initial GSED tools were designed using a data-driven paradigm. Data from over 70,000 children, assessed with 22 different instruments and using 2275 items were collated. A modified Rasch model was used to jointly model the data and select a subset of 807 items which showed promise of stability across countries. The psychometric performance of items is not sufficient for principled item selection and subject matter expert (SME) judgement data must be collected and synergised with the item parameters for robust item selection. The aim of this study was to create a novel, robust and reproducible methodology to collect and combine SME judgement about items with the psychometric properties of those items to streamline item selection for the creation of new global measurement tools. Methods: Eight internationally renowned SMEs in child development measurement provided inputs relating to three main areas. Firstly, data were collected on the extent to which groups of items measured the same behaviours. This was important so we could be cognizant of overlap between items in the final tool. Secondly, the domain of child development that each of the items measured was probed. We did this to ensure content validity in the final tool. Thirdly, global feasibility, relevance and appropriateness data was gathered for each item. Some items were clearly unsuitable for our purpose and an opportunity was given to the SMEs to flag those they felt did not meet the requirements to be included in the prototype for field testing. The Judgement data were combined with the psychometric data using an R-ShinyApp (online-dashboard) which facilitated the selection of the items for the final tool. Results & Conclusions: Two prototype tools were constructed to be tested in the field—the GSED Short Form, the caregiver reported tool, and the GSED Long Form the programmatic evaluation tool. The GSED is currently being validated gathering data from more than 8700 participants across seven countries**.** Disclosures: The authors declared no competing interests.

#### A17 Published https://doi.org/10.1007/s11136-021-02814-4

#### A18 Conducting a study in adult social care during a pandemic: lessons from the Measuring outcomes of people with dementia and their carers (MOPED) study

Barbora Silarova, Stacey Rand, Ann-Marie Towers, Karen Jones.

University of Kent, Canterbury, United Kingdom.

Background: In March 2020, the WHO declared the outbreak of the COVID-19 pandemic and subsequently, England started first of a series of country-wide lockdowns, adapted social distancing rules, restricted access to services and many, including researchers were asked to work from home. Aims: The aim of this paper is to summarise what we have learned about conducting a research with unpaid carers supporting people living with dementia during a COVID-19 pandemic. Methods: The aim of the MOPED study was to understand how well social care services support people living with dementia and their family and friends, who support them. The data collection started on 2^nd^ of March 2020 and the original pathways for recruitment of unpaid carers who support people with dementia included: adult social care services, carers’ and other local organisations, the Join Dementia Research register and social media strategy. The aim was to collect data through questionnaire either as a paper or online version. Results: Recruitment through adult social care services had to be completely halted. As a response to this, we engaged with NHS sites to support recruitment to our study as part of the RESTART (launched in May 2020). Distribution of study questionnaires through carers’ and other local organisations was also not possible. While some organisations promoted the study via their social media and e-newsletters, this strategy has not resulted in any recruits, similar to promoting study via departmental social media accounts. More targeted approaches, e.g. contacting participants individually via NHS or Join Dementia Research, were more successful. Conclusions: Conducting a study in the context of adult social care during a COVID-19 pandemic has been challenging. The successful delivery of the study has required a flexible approach to data collection and exploring recruitment pathways not considered at the start of the study. Disclosures: The authors declared no competing interests.

### Session 3a: Social Care.

#### A19 Measuring social-care related quality of life of people with dementia who are unable to self- report and their carers

Stacey Rand, Barbora Silarova, Ann-Marie Towers, Karen Jones.

University of Kent, Canterbury, United Kingdom.

Background: People living with dementia are a growing subgroup of social care service users. Two-thirds of people with dementia live in their own homes and may use community-based social care services to maintain their QoL and also the QoL of informal carers. Therefore, it is important that data used to evaluate services and to guide policy-making considers the views of people with dementia and their carers. The Adult Social Care Outcomes Toolkit (ASCOT) is a self-report measure of social care-related QoL developed for social care research, evaluation and monitoring. Among other adapted data collection methods, a proxy-report version (ASCOT-Proxy) has been developed for use when someone is unable to self-report. Alongside the ASCOT measures for service users, there is also a version for carers (ASCOT-Carer). While the measurement properties of the ASCOT-Carer has been established with a heterogeneous sample of carers in England, they have yet to be evidenced specifically with carers of people with dementia. Aims: To establish the psychometrics properties of the ASCOT-Proxy and ASCOT-Carer in a sample of carers of community-based social care service users with dementia, unable to self-report. Methods: A survey of up to 300 carers of people with dementia will collect data using the ASCOT-Proxy and ASCOT-Carer and related QoL measures (e.g. EQ-5D), as well as individual characteristics. Construct validity will be evaluated by hypothesis testing, structural validity by factor analysis, and Internal reliability by Cronbach’s alpha. Response rates, response distribution and missing data will be used as indicators of feasibility. Results: The data collection is ongoing (until March 2021). Preliminary analysis indicates that ASCOT-Proxy and ASCOT-Carer are feasible, valid and reliable measures of SCRQoL. Conclusions: By establishing the measurement properties of these tools, we further the development of tools available for use in social care research that may guide policy and practice. Disclosures: The authors declared no competing interests.

#### A20 Published https://www.britishgerontology.org/public/31949/BSG_Conference_Book_2021_Online_v2.pdf

#### A21 PROMs in social care: Preliminary findings from a scoping review.

Sarah Hughes^1^, Olalekan Aiyegbusi^1^, Daniel Lasserson^2^, Philip Collis^1^, Samantha Cruz-Rivera^1^, Christel McMullan^1^, Grace Turner^1^, Jon Glasby^1^, Melanie Calvert^1^.

^1^University of Birmingham, Birmingham, United Kingdom. ^2^University of Warwick, Coventry, United Kingdom.

Introduction: In social care, self-reported outcome measures are used routinely to collect data relating to social-care related quality of life and well-being as part of aggregate-level surveys. However, it is unclear how self-report measures are used with individuals to help them manage their own care and support. People with care and support needs often live with complex, long-term health conditions. For this reason, patient-reported outcome measures (PROMs), as measures of health and wellbeing, could help support individuals who are direct users of social care. Aims: The aim of this scoping review is to explore the evidence relating to the use of PROMs with adults who use care and support services. Methods: Medline (Ovid), PsychInfo (Ovid), ASSIA (Proquest), Social Care Online (SCIE), Web of Science, and EMBASE (Ovid) were searched systematically to identify sources of evidence published since 2010. Electronic database searching was supported by grey literature and hand searches. The Critical Appraisal Skills Programme (CASP) and AACODs checklists were used to appraise the quality of sources. Data charting extracted relevant data into tables and an analysis and synthesis of qualitative data was undertaken using the Framework Method. Results: This review is ongoing. In this presentation, preliminary findings from data charting and qualitative analysis will be shared. A description of PROMs used by individuals who use care and support services and evidence relating to their effectiveness, barriers and facilitators to their implementation, and stakeholders’ perspectives on their use will be presented. Conclusions: To our knowledge, this scoping review will be the first review to provide an overview of the evidence around the use of PROMs with adults who are direct users of care and support services. Review findings will help to establish the relevance, acceptability and feasibility of using PROMs in adult social care, informing directions for future research. Disclosures: SEH is supported by the National Institute of Health Research (NIHR) Applied Research Centre (ARC), West Midlands and Innovate UK (part of UK Research and Innovation) and receives personal fees from Aparito Limited and Cochlear Limited outside of the submitted work. MC is a National Institute for Health Research (NIHR) Senior Investigator and receives funding from the National Institute for Health Research (NIHR) Birmingham Biomedical Research Centre, the NIHR Surgical Reconstruction and Microbiology Research Centre and NIHR ARC West Midlands at the University of Birmingham and University Hospitals Birmingham NHS Foundation Trust, Health Data Research UK, Innovate UK (part of UK Research and Innovation), Macmillan Cancer Support, UCB Pharma. MC has received personal fees from Astellas, Takeda, Merck, Daiichi Sankyo, Glaukos, GSK, and the Patient-Centered Outcomes Research Institute (PCORI) outside the submitted work. OLA is supported by the National Institute of Health Research (NIHR) Birmingham Biomedical Research Centre (BRC), Birmingham, West Midlands. OLA also receives funding from the Health Foundation and has received personal fees from Gilead Sciences. DL is supported by the NIHR ARC West Midlands and the NIHR Community Healthcare MedTech and IVD Cooperative, hosted by Oxford Health NHS Foundation Trust. JG is a non-executive director of University Hospitals Birmingham, adjunct professor at Curtin University, member of the leadership team for an ESRC large grant on sustainable adult social care, and member of the expert advisory group for West Midlands ARC. CM receives personal fees from Aparito Limited outside of the submitted work. PC, GT, and SCR have no conflicts of interest to declare.

### Session 3b: Patient, Public Involvement and Engagement (PPIE)

#### A22 Adapting a patient reported questionnaire to diagnose wound infections after surgery around the world: Global experience with Community Engagement & Involvement in Low-and Middle-income countries (Lay abstract).

James Glasbey, NIHR Global Health Research Unit on Global Surgery, University of Birmingham, Birmingham, United Kingdom.

Background: An infection in the cut (‘wound’) made in the tummy during surgery is the most common problem that patients face during their recovery from an operation. Correct diagnosis of an infection after a patient leaves the hospital is important, both to keep them safe at home and for research purposes. Challenge: Traditionally, surgeons ask patients to travel back to the hospital after their operation in order to make a diagnosis. However, in poorer countries this can be a real challenge for patients, who may have to travel many miles to their hospital and face unnecessary costs. What is more, unnecessary visits to hospital during the COVID-19 pandemic may be a risk to their safety. Methods: This year, I have worked with patients, research staff and surgeons from around the world to translate a specialised questionnaire (a patient reported outcome measure) which can be completed over the telephone to diagnose wound infection into ten languages. This has followed expert recommendations for working with patients from several countries. Working with the community, I have adapted the questionnaire to check that it captures patients’ experiences across different cultures – from rural Rwanda to urban cities in India. Outputs: Together with patients, we have designed a training package for researchers to make the experience as acceptable and successful as possible for patients. To ensure broad inclusion of patients from different cultures, genders and backgrounds, we have adapted to use video-chat to discuss the project during COVID-19. Future work: We have now begun a study across seven countries to test how accurate the adapted questionnaire is for diagnosis of infection. The overall aim is to create a pathway to identify problems after surgery early, without the need to travel back to hospital to see a doctor. Disclosures: The authors declared no competing interests.

#### A23 Published https://doi.org/10.1007/s11136-021-02976-1

#### A24 Patient Reported Outcome Measures (PROM) for Abdominal Aortic Aneurysm (AAA) repair Pre-operative and Post-Operative.

Louise Patullo, Anthony Jaipersad.

University Hospitals of North Midlands, Stoke-on-Trent, United Kingdom.

Aims: It has been noted that data relating to improving practice and providing patient-centred care, involving patients in the decision-making process is not readily available. One of the postulations for this is the inability to highlight the importance of Patent Reported Outcome Measures (PROM’s) alongside clinical outcome measures. This study seeks to prospectively assess the need for PROM’s for elective and emergency Abdominal Aortic Aneurysm surgery. Methods: Prospective qualitative audit of 50 randomly selected vascular patients across 4 sites. Pre-operative questionnaires were provided at the pre-assessment clinic in both the vascular hub and spoke sites. Post-operative questionnaires were sent to the patient at least 4 weeks post-operation and collected at the patient’s next outpatient appointment. Results: 50 questionnaires were returned, 62% reported having an Open Aneurysm Repair (OAR), 30% EndoVascular Aneurysm Repair (EVAR) surgery. A total of 36% of patients reported discussing both OAR and EVAR surgery. Complications were recorded in 18% of patients related to their surgery. However, 32% of patients did not feel their recovery met their expectations. Expectations not met were: scars, time to recovery, long-term effects, ongoing pain and further complications. In the post-operative group, 32% of patients felt a more explanatory patient information leaflet would have better helped their understanding of what to expect. Conclusions: Despite continuing effort in the NHS towards transparency, this study identifies that patient communication remains a major issue in patient dissatisfaction. Patients require clear written and verbal information presented which they can understand, putting them at the centre of their care. Limitations in the questionnaires highlighted in our study provided difficulty in leading to a change in practice. A validated questionnaire with an interpretation of results needs to be used to encourage a change in practice and demonstrate the importance of PROMS and patient-centred care. Disclosures: The authors declared no competing interests.

### Session 3c: Psychometrics/Item Response Theory

#### A25 Computerised Adaptive Testing for the Oxford Hip and Knee Scores Dramatically Reduces the Number of Items Needed Without Compromising Precision

Jonathan Evans^1^, Chris Gibbons^2^, Jose Valderas^1^.

^1^University of Exeter Medical School, Exeter, United Kingdom. ^2^The University of Texas MD Anderson Cancer Center, Texas, USA.

Aims: To apply modern psychometric analysis and computerised adaptive testing (CAT) simulations to the world’s largest repository of pre-operative hip and knee Patient-Reported Outcome Measures (PROMs). Methods: The 2018/19 National PROMs programme 12-item Oxford Hip and Knee Scores (OHS and OKS), recorded before a primary joint replacement, were evaluated for dimensionality, local dependency, item fit and marginal reliability. A Graded Response Model utilised to develop item-specific difficulty and discrimination parameters. A CAT simulation was undertaken to assess item responses within the full-length administration and CAT counterparts at varying degrees of precision (Standard Error). Average CAT length and the correlation between full-length and CAT scales were calculated. Results: IRT analysis was conducted on 40,432 OHS and 44,714 OKS observations. Primary OHS and OKS were both unidimensional (Root Mean Square Error of Approximation (RMSEA) of 0.08 and 0.07 respectively). Marginal reliability (MR) was 0.914 and 0.903 respectively. Primary CAT at 90% and 80% precision required a median of 3 items (range 3–12)(r = 0.97) and median 2 items (2–3)(r = 0.95) respectively for the OHS, and median of 4 items (3–12)(r = 0.98) and 2 items (2–4)(r = 0.94) for the OKS. Conclusions: We have established that the application of IRT to the OHS and OKS produces an efficient and substantially reduced CAT. By reducing the number of items required and applying a standardised measurement scale, we have demonstrated a path to increases compliance and interpretability, whilst reducing patient and institutional burden for these ubiquitous outcome measures. Disclosures: The authors declared no competing interests.

#### A26 Published: https://sapc.ac.uk/doi/10.37361/asm.2021.1.1

#### A27 Comparing the EQ Health and Wellbeing (EQ-HWB) instrument with other measures in different populations

Wael Mohammed, Clara Mukuria, John E Brazier.

The University of Sheffield, Sheffield, United Kingdom.

Background: Generic preference-based health measures (PBM) have limitations in some conditions while different measures may be required for other sectors such as social care which limits comparability. The EQ-HWB was developed to capture a broad range of outcomes across different populations and enhance cross-sector comparisons. Aims: This study aimed to assess the construct validity of the EQ-HWB (25-items) and the EQ-HWB-S (9-items), and compare them to the EQ-5D-3L, EQ-5D-5L, the Short Warwick Edinburgh Mental Wellbeing Scale (SWEMWBS) and the Adult Social Care Outcomes Toolkit (ASCOT). Methods: Secondary data from patients and the general population from the UK was used (N = 1755). Transformed (0–1) sum-scores for all the measures were compared to the EQ-5D visual analogue scale (EQ-VAS) and the SWEMWBS to assess construct validity. Convergent validity was evaluated using Spearman rank-order correlation and Lin's concordance correlation coefficients. Whereas, known group assessment was established by estimating the effect sizes. Sub-group analysis was undertaken for informal carers and patients who reported a mental health condition. Results: The mean and distribution of the EQ-HWB [EQ-HWB: 0.66 ± 0.21,EQ-HWB-S: 0.63 ± 0.24] was similar to EQ-VAS [0.64 ± 0.25] and the other measures. All measures were correlated to and showed high agreement levels with EQ-VAS. EQ-HWB and EQ-HWB-S had stronger associations with SWEMWBS among informal carers and those with mental health problems than the EQ-5D versions. The EQ-HWB and EQ-HWB-S had larger effect sizes, 1.1 [1, 1.2] and 1.1 [1, 1.2], compared to EQ-5D-3L 0.7 [0.6, 0.8], EQ-5D-5L 0.6 [0.5, 0.7], ASCOT 0.9 [0.8, 1] and SWEMWBS 0.9 [0.8, 1] in patients with mental health. Conclusions: Overall, the results suggest that the EQ-HWB reflects health and wellbeing and better reflects known differences in mental health and informal carers than other measures. Future work comparing utility values and in other populations is needed. Disclosures: The authors declared no competing interests.

